# Vaccination Eruptions

**Published:** 1893-04-29

**Authors:** 


### THE PRACTITIONERS' BOOKSHELF.

### VACCINATION ERUPTIONS.*

There are two parts of this little book which are very unsatisfactory; and these parts happen, unfortunately, to be the preface and the introduction. In the few pages of prefatorial and introductory matter Dr. Poole indulges in some weak generalisations, ungrammatically expressed, and thereby produces a very undesirable condition of irritation in the reader's mind. Before we come to the actual book itself we have decided from the preface that it must be feeble, and inconsequent, as well as illogical. But the proper quality of the matter, when it has been fairly reached, is the very opposite of all this. It is solid, interesting, well and clearly expressed, and constitutes a very valuable contribution to an important subject. When a new edition is asked for, as it will be, Dr. Poole will be well advised to write a new preface and introduction, or dispense with these superfluities altogether.

The whole question of vaccination is of the greatest possible importance at the present time. It is the keystone of that modern arch of medical treatment which is being reared on a microbial pathology. Manifestly, therefore, vaccination should be studied in all its moods and tenses, in every conceivable aspect of its normal and its abnormal manifestations. Dr. Poole's little book is a contribution of facts, of facts intelligently comprehended, and set forth for the reader's consideration in due and judicial order. Most interesting, and even surprising, some of the facts are. Under the heading of "Late appearance of Vesicles" for example, we are told not only that the vaccine vesicle may sometimes be much longer in making its appearance than the normal two or three days, but that it may be delayed for a period of months, or even years. The most extraordinary case of this kind is one recorded by Sir Thomas Watson. In this case the vesicles did not appear for fourteen years after the date of inoculation. The patient was a girl who, having been vaccinated as a baby, had never "taken." At the age of fourteen she was attacked with influenza, and under the physiological stimulus of this disease pains in the arms, at the points of vaccination, began to be felt, and in a short time perfectly normal vaccine vesicles were developed, vesicles so perfect that an elder sister was re-vaccinated from them, and with the most complete success.

Under the heading of "Accidental Vaccination" a very curious case is mentioned. A gentleman, aged 28, who had chafed himself with riding, applied vaseline from a pot which his wife was using for dressing the vaccinated arm of her child. In a very short time a fine crop of well-matured vaccine vesicles appeared on the buttocks, to which the vaseline had been so unsuspectingly applied. Potent stuff this vaccine lymph, and absolutely constant in its constitution!

"There is a stage in the study of vaccination," says Dr. Poole, "when one is inclined to become anti-vaccinist, to join the rabble, and to share their bubble glory." Not a doubt of it; and anti-vaccinators would do well to ponder such a state of mind in medical men. Hundreds of medical men, in whom the strongest passion is that of devotion to the well-being of their fellow men, have commenced the special study of vaccination under the influence of the exciting appeals of anti-vaccinators. It is a striking fact that of those hundreds not so many as one per cent. ever became anti-vaccinators themselves. A wider and profounder acquaintance with facts convinces them that, notwithstanding the plausible objections which can be made against vaccination in the regions of sentiment and theory, the substantial and monumental truth remains, that whereas before vaccination small-pox was the terror of civilisation, since the inauguration of Jenner's prophylaxis small-pox is like a ghost, which intelligence knows to be laid and fears no more.

It is not possible in the space at our disposal to give more than a sample or two of the class of questions raised in the book, and of the interesting facts and cases by which the arguments are illustrated. But the monograph, which consists of no more than 120 pages, is of real importance, and will be recognised as a valuable aid to the elucidation of some of the most significant questions which arise out of vaccination. It ought to be read, and no doubt will be read by vaccinators and anti-vaccinators alike. We congratulate Dr. Poole on his work, always of course excepting his preface and his introduction.

* "Vaccination Eruptions." By T. D. Poole, C.M. (Edinburgh: E. and S. Livingstone.)

### ELEMENTARY CHEMISTRY.

In his Lecture Course in Elementary Chemistry* Mr. Lilley has succeeded admirably in carrying out his self-imposed task to provide "a short and clear statement of the most important results of chemical science." The volume, though small, contains a great deal of information, numerous lustrations, and some well-chosen exercises and questions with answers to the numerical examples and a complete index. We can confidently recommend this Lecture Course to all who wish to gain an intelligent, if elementary, knowledge of the principles underlying chemical science.

* "A Lecture Course in Elementary Chemistry." By H. T. Lilley, (Simpkin, Marshall, Hamilton, Kent, and Co., London. Price 2s. 6d.)

